# Impact of codeine rescheduling on prescribing of codeine and other opioids: Interrupted time series analyses using Australian general practice data

**DOI:** 10.1111/bcp.16246

**Published:** 2024-09-19

**Authors:** Helena Cangadis‐Douglass, Ting Xia, J. Simon Bell, Suzanne Nielsen

**Affiliations:** ^1^ Monash Addiction Research Centre, Eastern Health Clinical School Monash University Melbourne VIC Australia; ^2^ Centre for Medicine Use and Safety, Faculty of Pharmacy and Pharmaceutical Sciences Monash University Melbourne VIC Australia; ^3^ Department of Epidemiology and Preventive Medicine, School of Public Health and Preventive Medicine Monash University Melbourne VIC Australia

**Keywords:** Australia, codeine, interrupted time series analysis, opioid, prescribing patterns, rescheduling

## Abstract

**Aims:**

We aimed to determine the impact of codeine rescheduling on prescribing of codeine and other opioids, with a focus on demographic and diagnoses associated with codeine prescribing before and after rescheduling of codeine to prescription‐only in February 2018.

**Methods:**

We used interrupted time series analysis (February 2016‐February 2020) and probit regression to examine prescribing of codeine and other opioids according to primary care data from 464 general practice clinics in Victoria, Australia.

**Results:**

The rate of codeine prescribing increased in the month following rescheduling (additional 76 people/10000, 95% confidence interval [CI] 49‐103), then declined to baseline rates (slope −2.02, 95% CI 3.79, −0.25). Prescribing of other opioids did not change. Post rescheduling, females were more likely to receive codeine prescriptions compared to males (β = 0.094, 95% CI 0.08‐0.108) and those aged 70‐79 years were more likely to receive codeine compared to those aged <30 years. Those residing in the least disadvantaged areas had a greater probability of being prescribed codeine than those in more disadvantaged areas after rescheduling (β = 0.154, 95% CI 0.129‐0.179). A documented mental health diagnosis (β = 0.067, 95% CI 0.052‐0.082) or migraine diagnosis (β = 0.057, 95% CI 0.037‐0.078) was associated with increased likelihood of receiving a codeine prescription after rescheduling compared to before in contrast to those without such a diagnosis.

**Conclusion:**

Codeine rescheduling did not result in a sustained increase in codeine prescribing nor a change in the prescribing of other opioids. Patient factors associated with increased codeine prescribing after compared to before rescheduling included female sex, older age, migraine diagnosis and comorbid mental health conditions.

**Registration:**

EU PAS Register (EUPAS43218).

**What is already known about this subject**

- Opioid‐related harm in Australia has been growing, and codeine comprises a large proportion of pharmaceutical opioids use.
- Codeine has been associated with a wide range of harms, including codeine dependence.
- In February 2018 all codeine products became prescription medicines in Australia, with low‐dose products no longer available over the counter, but few studies have examined the impact of this change on overall codeine prescribing.

**What this study adds**

- Codeine rescheduling did not result in a sustained increase in codeine prescribing or a change in the prescribing of other opioids.
- Some characteristics (female sex, older age, migraine diagnosis and comorbid mental health conditions) were associated with new codeine prescriptions after the policy change.

## 1 INTRODUCTION

Opioid‐related harm in Australia has been a growing concern.^1^, ^2^ Between 2013 and 2017, 3 million adults used and over 1.9 million adults initiated opioids each year.^3^ Australia was ranked the seventh highest country globally for consumption of opioids between 2017 and 2019.^4^

Codeine has historically comprised a large proportion of pharmaceutical opioid use in Australia. In 2011, codeine represented 41% of all opioids dispensed nationally in defined daily doses/1000 population/day.^5^ From 2012 to 2016, codeine‐containing products represented over 26% of opioid prescriptions in general practice^6^ and they accounted for two‐thirds of opioid pack sales nationwide in 2013.^7^

Codeine has been associated with a wide range of harms, including opioid dependence, demonstrated by an increase in individuals seeking opioid agonist therapy for codeine dependence in Australia. Nationally in 2017, 4.2% of people receiving pharmacotherapy for opioid dependence reported codeine as their primary drug of dependence, increasing from 2.7% in 2014.^8^ Codeine‐related fatalities increased from 3.5 to 8.7 deaths per million population between 2000 and 2009,^9^ with accidental overdoses accounting for a higher proportion of deaths (48.8%) compared to intentional deaths (34.7%), and this percentage rising overtime. Codeine combination products, such as codeine‐ibuprofen and codeine‐paracetamol have been associated gastrointestinal disease, renal failure, medication overuse headache and severe hypokalemia.^10^, ^11^, ^12^, ^13^ The risks of these harms are increased in people with prolonged and high‐dose usage.^11^

Prior to February 2018 codeine was available from Australian pharmacies without a prescription. In response to increasing harms associated with codeine, the Therapeutic Goods Association (TGA) rescheduled codeine from an over‐the‐counter pharmacy only (Schedule 2) or pharmacist only (Schedule 3) medication to a prescription‐only (Schedule 4) medication.^14^ This meant that all codeine products required a prescription.

Research has suggested that codeine rescheduling was associated with reduced pharmacy sales,^15^ emergency department presentations^16^ and codeine‐related poison centre calls.^17^ Studies suggest rescheduling did not increase use among regular codeine users^18^ or result in substitution for stronger opioids.^19^ The latter study,^19^ which examined Pharmaceutical Benefits Scheme (PBS) dispensing claims data, found no increase in the prescribing of subsidized codeine, but was not able to consider private prescriptions. In the Australian context, much of the existing evidence on opioid use relies on data derived from PBS dispensing claims data. The PBS offers subsidized medications for Australian citizens, permanent residents and visitors from countries with reciprocal healthcare agreements. However, in 2014 pharmaceutical dispensing data underestimated national opioid utilization by up to 12.4% in Australia^20^ and this percentage is likely to have increased in recent years with the large increase in prescribing of non‐subsidized opioids like some tapentadol formulations^21^ and prescribing of unsubsidised low‐dose codeine products. Therefore, utilizing prescribing data addresses a limitation of datasets like the PBS by including both private and subsidized medications.

Codeine remained the most commonly misused opioid among individuals engaging in non‐medical use of opioids in 2019, with 61% of people reporting misuse of an opioid specifically reporting use of codeine.^22^ One study reported an increase in codeine‐paracetamol prescribing in Australian war veterans and their dependents following rescheduling.^23^ However, no studies beyond the single study using aggregated PBS data^19^ have examined rates of codeine and other opioid prescribing before and after rescheduling in the general population.

In Australia, more patients were dispensed codeine than other opioids in 2021.^2^ More than half of all opioids prescribed in Australia are from general practitioners.^3^ Recent studies show codeine accounted for more than half (52%) of all opioid private‐market prescriptions between 2013 and 2018 in Australian general practice.^24^ Given this high use, understanding the impact of codeine rescheduling is crucial for optimizing prescribing practices.

The objective of this study was to investigate the impact of codeine rescheduling on prescribing of codeine and other opioids, with a focus on demographic and diagnostic factors associated with codeine prescribing before and after rescheduling.

## 2 METHODS

### 2.1 Study design and data source

We analysed electronic medication record data from general practice clinics in the south‐eastern region of Victoria, Australia. Victoria is Australia's second most populous state and comprises 26% of the Australian population.^25^ Data were sourced from the POpulation Level Analysis and Reporting (POLAR) dataset with approval from three primary health networks (PHNs) in Victoria.^26^ The GP practice clinics encompassed metropolitan and regional areas from the Eastern Melbourne, South Eastern Melbourne and Gippsland PHNs, which cover 48% of Victoria's population.^27^ Data were collected from 464 general practitioner (GP) practices, representing 45% of total number of GP practices located in these three PHNs. The study design, data source, data clean strategy, data analysis plan and cohort profile are outlined in the 'Using primary care data to understand Opioid Prescribing Policy Impacts and Clinical Outcomes' (OPPICO) study protocol and cohort profile paper.^27^, ^28^ The reporting of the study adhered to the REporting of studies Conducted using Observational Routinely‐collected health Data statement for PharmacoEpidemiology (RECORD‐PE).^29^

### 2.2 Patients

We extracted data on patient demographics, diagnoses and prescribed medications from 1 February 2016 to 31 January 2018 (2 years prior to rescheduling – baseline period) and from 1 February 2018 to 31 January 2020 (2 years after rescheduling – observation period). All patients aged 14 years and older prescribed an opioid analgesic from 1 February 2016 to 31 January 2020 were included. Patients were included if they had a recorded activity (eg, a face‐to‐face consultation, telephone consultation or vaccination) over the study period (active patients). Patients with a documented cancer diagnosis at any point during the study period were excluded. Full patient demographic details, including concession status, socioeconomic status and geographical remoteness, are extensively defined in the OPPICO study protocol^28^ and cohort profile paper.^27^

### 2.3 Outcomes

We separately examined changes in monthly rates of codeine and other opioid prescriptions before and after rescheduling compared to other opioids. We also investigated comorbidities of interest (such as mental health diagnosis) and conditions associated with codeine prescribing before and after the scheduling, such as diagnosis of back and neck pain, migraine, arthritis/rheumatism and musculoskeletal conditions. All opioid analgesics marketed in Australia were included in this study. Opioids were identified through generic name and the World Health Organization's Anatomical Therapeutic Chemical (WHO ATC) code N05A (Table 1).^30^ The number of patients in the POLAR cohort varied over time, so monthly prescription rates were calculated to account for the differences in the denominator. Diagnoses derived from the patients' historical medical records and were coded using a validated approach by Outcome Health using Systematized Medical Nomenclature for Medicine ‐ Clinical Terminology (SNOMED CT) codes.^26^ Further details of the SNOMED codes used for diagnosis identification are comprehensively documented in the published protocol paper.^28^

**TABLE 1:** Opioids included in the study

Opioid	ATC code
***Codeine (treatment group)***	
Codeine and codeine combinations	R05DA04, N02AJ06, N02AJ07, N02AJ08, N02AJ09
***Other opioids (comparison group)***	
Buprenorphine	N02AE01
Dextropropoxyphene	N02AC04
Fentanyl	N02AB03
Hydromorphone	N02AA03
Methadone	N02AC52
Morphine	N02AA01
Oxycodone	N02AA05
Oxycodone‐naloxone	N02AA55
Pethidine	N02AB02
Tapentadol	N02AX06
Tramadol and tramadol combinations	N02AX02, N02AJ13

### 2.4 Data analysis

Data were summarized using frequencies and proportions. If prescriptions included repeats, we generated additional records to incorporate subsequent supplies into the calculations. Resupply dates for repeats were estimated: 28 days for slow‐release opioids or patches, and 14 days for other opioids. The number of repeats for a given prescription was capped at 12 (affecting less than 0.01% of outliers) to account for assumed data errors from unusually high values. Monthly rates of codeine prescribing and other opioids were examined per 10 000 active patients (patients with recorded activity within the month of reporting) in the whole POLAR primary care cohort in the same month. Interrupted time series (ITS) analysis was used to examine immediate (step) changes and trend (slope) changes in codeine prescribing following rescheduling. In total, 48 monthly data points were included in the regression model, including 24 monthly data points before (pre‐rescheduling period) and 24 monthly data points after (post‐rescheduling period) rescheduling. Monthly rates of other opioid prescribing were included as a comparison group. Changes were evaluated with segmented linear regression models using the ordinary least squares method with Newey‐West standard errors adjustment. The Cumby‐Huizinga test^31^ was used to examine autocorrelation between time periods. Harmonic terms were added to the models to account for seasonality. Lay effect and seasonality terms that were significant (*P* < .05) were retained in the final models. As the scheduling decision was announced by TGA in December 2016, a sensitivity analysis was performed to examine the impact of this additional announcement period on codeine prescribing.

We utilized probit regression analysis to investigate how demographics (eg, age, gender and social economic status) and clinical characteristics (eg, mental health and type of pain conditions) affected prescribing codeine before and after codeine rescheduling. The independent variable, having a codeine prescription only recorded in the 2 years after codeine rescheduling, was coded as a binary outcome. The models were adjusted for relevant covariates, including age, gender and comorbidities. *P* values <.05 were deemed statistically significant. Analyses were performed using Stata/MP 17.^32^

### 2.5 Ethics and consumer involvement

The research question was identified by a stakeholder group including healthcare providers and consumers.^33^ Ethical approval was obtained from the Monash University Human Research Ethics Committee (#24139).

### 2.6 Nomenclature of Targets and Ligands

Key protein targets and ligands in this article are hyperlinked to corresponding entries in http://www.guidetopharmacology.org, the common portal for data from the IUPHAR/BPS Guide to PHARMACOLOCY, and are permanently archived in the Concise Guide to PHARMACOLOGY 2019/20.^62^, ^63^

## 3 RESULTS

### 3.1 Trends in monthly prescribing rate before and after codeine rescheduling

**FIGURE 1:**
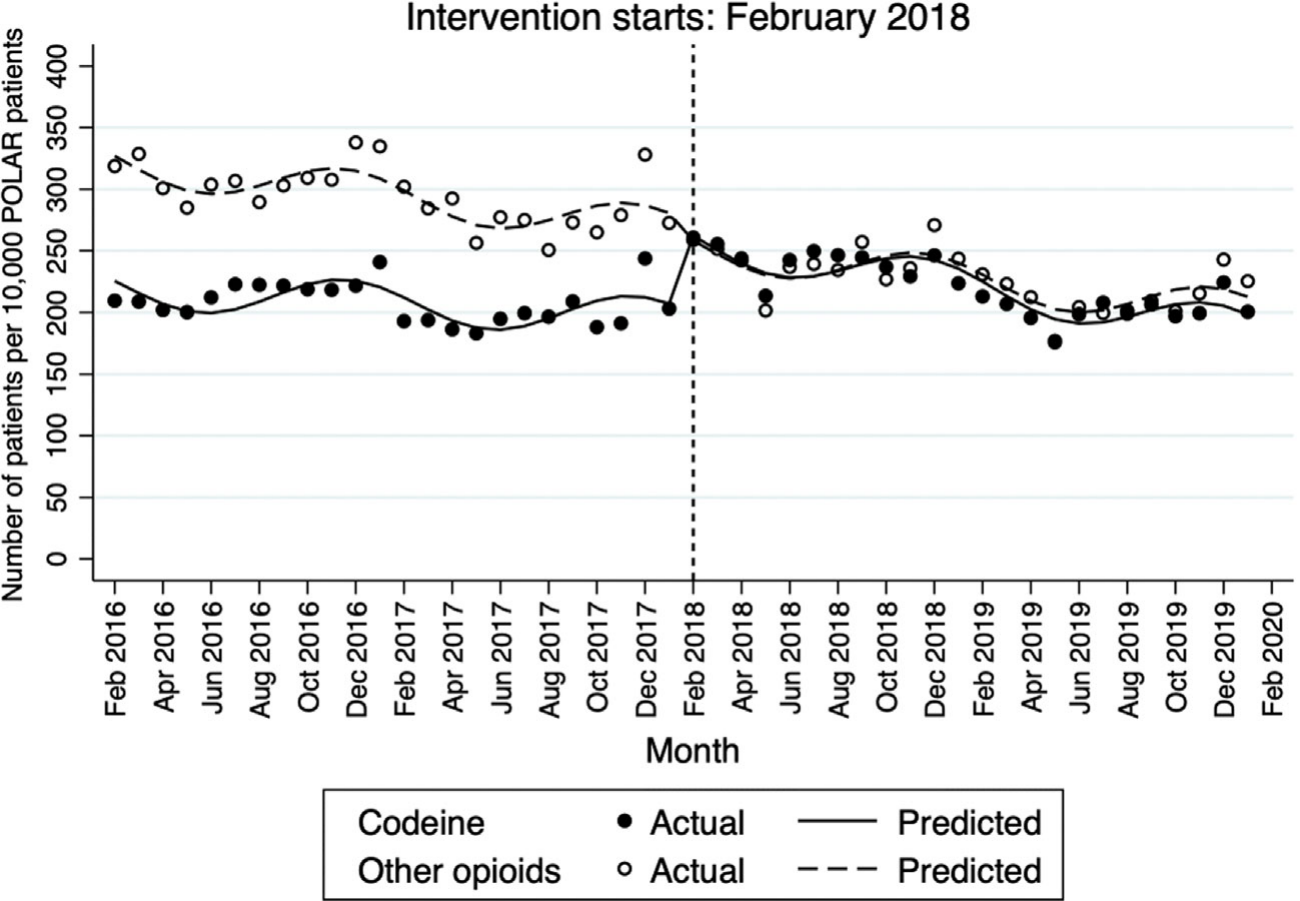
Rates of patients who had codeine and other opioid prescriptions per 10 000 POLAR patients (ie, the whole primary care cohort). Solid line: predicted trend based on the seasonally adjusted interrupted time series model.

From February 2016 to January 2020, 139 943 individuals were prescribed codeine. Of these, 74 818 were prescribed codeine prior to rescheduling and 65 165 people were prescribed codeine only after rescheduling. Figure 1 depicts the trend of monthly prescribing of codeine and other opioids per 10 000 patients.

From February 2016 to January 2018, the monthly rate of opioid prescriptions decreased (reducing by 2.23 patients per 10 000 patients per month, 95% confidence interval [CI] −3.37, −1.30; Table 2).

**TABLE 2:** Effect of codeine rescheduling implementation on rates of opioid prescribing per 10 000 POLAR primary care cohort

	ß (95%CI)
**Pre‐intervention**	
Baseline slope of the monthly prescribing rates	**−2.23 (−3.37, −1.30)**
Initial mean level difference in the monthly prescribing rates (codeine *vs* other opioids)	**−101.51 (−116.31, −86.71)**
Difference in the mean baseline slope in the monthly prescribing rates (codeine *vs* other opioids)	1.21 (−0.19, 2.63)
**Post‐intervention**	
Immediate level change in the monthly prescribing rates following codeine rescheduling (other opioids)	−12.68 (−33.07, 7.71)
Post‐intervention slope change in the monthly prescribing rates following codeine rescheduling (other opioids)	0.03 (−1.31, 1.37)
Immediate level change in the monthly prescribing rates following codeine rescheduling (codeine *vs* other opioids)	**76.25 (49.26, 103.24)**
Post‐intervention slope change in the monthly prescribing rates following codeine rescheduling (codeine *vs* other opioids)	**−2.02 (−3.79, −0.25)**

*Note*: Bold text indicates significant results.

Following codeine rescheduling in February 2018, an initial increase (step change) in the rate of patients receiving codeine prescriptions was observed, with an increase of 76.25 patients per 10 000 POLAR patients immediately following rescheduling (95% CI 49.26, 103.24) relative to the rate of patients receiving other opioid prescriptions. The step change in the rate of other opioids prescribing following codeine rescheduling was not significant (β = −12.68, 95% CI −33.07, 7.71). In the 24 months following rescheduling, there was a significant decreasing trend (slope change) in the rate of codeine prescriptions compared to other opioid prescriptions (−2.02 fewer patients per 10 000 patients per month, 95% CI −3.79, −0.25). No significant trend changes in the monthly rate of patients receiving other opioid prescriptions were observed post intervention (β = 0.03, 95% CI −1.31, 1.37).

In the sensitivity analysis that included an additional period corresponding to the announcement of the codeine rescheduling in December 2016, we noted that there was no immediate (step) change (β = ‐2.241, 95% CI −25.152.129‐20.670) or longer‐term (slope) change (β = −0.500, 95% CI −3.786‐2.786) in the monthly rate of codeine prescribing following the announcement.

### 3.2 Characteristics of people prescribed codeine before and after codeine rescheduling

Table 3 shows the characteristics of people prescribed codeine before and after codeine rescheduling. The probit regression analysis revealed several significant associations between demographic and clinical factors, and the probability of being prescribed codeine prescriptions post‐rescheduling. The probability of being prescribed codeine prescriptions after the rescheduling increased by 9.4% (95% CI 0.081‐0.108) in females compared to males. The probability of being prescribed codeine after the rescheduling increased by age, with the 70‐79 years age group probability increased by 11.2% (95% CI 0.082‐0.143) compared to those aged <30 years. People with a Department of Veterans' Affairs (DVA) or healthcare and pension concession status had a lower probability of being prescribed codeine after rescheduling (DVA β = −0.146, 95% CI −0.267 to −0.025, healthcare and pension concession β = 0.055, 95% CI −0.072 to −0.039) compared to those without a DVA or healthcare concession card. Following rescheduling, those who resided in the least disadvantaged areas had a 15.4% (β = 0.154, 95% CI 0.129‐0.179) greater probability of being prescribed codeine prescriptions than those residing in more disadvantaged areas. Compared to patients living in the major cities, the probability of being prescribed codeine after rescheduling decreased by 5.1% in patients who lived in the inner regional areas (β = −0.051, 95% CI −0.072 to −0.039).

**TABLE 3:** Demographic and clinical‐related factors associated with receiving a codeine prescription post codeine rescheduling.

	Prior to codeine rescheduling, N (%), (N = 74 818)	After codeine rescheduling, N (%) (N = 65 125)	Coefficient (95% CI)
**Sex**			
Male	33 568 (44.9)	26 544 (40.8)	Reference
Female	41 250 (55.0)	38 581 (59.2)	**0.094 (0.081‐0.108)**
Other/not specified^#^	127 (0.15)	42 (0.05)	**‐**
**Age (years)**			
<30	10 861 (14.5)	8878 (13.8)	Reference
30‐39	15 646 (20.9)	12 564 (19.3)	−0.014 (0.039, 0.007)
40‐49	14 253 (19.0)	12 486 (19.2)	**0.036 (0.013, 0.060)**
50‐59	13 149 (17.6)	11 743 (18.0)	**0.046 (0.022, 0.070)**
60‐69	9.821 (13.1)	9, 384 (14.4)	**0.100 (0.074, 0.126)**
70‐79	6707 (9.0)	6331 (9.7)	**0.112 (0.082, 0.143)**
80+	4507 (6.0)	3679 (5.7)	0.024 (−0.0.011, 0.060)
**Pension status**			
Seniors health card	249 (0.3)	261 (0.4)	0047 (−0.063, 0.157)
DVA card	249 (0.3)	179 (0.3)	**−0.146 (−0.0.267, −0.025)**
Healthcare and pension concession	23 509 (33.0)	19 541 (30.0)	**−0.055 (−0.072, −0.039)**
Not specified	50 937 (68.0)	45 184 (69.3)	Reference
**Remoteness**			
Major cities	60, 680 (81.0)	54, 187 (83.2)	Reference
Inner regional	13 159 (17.6)	10 126 (15.5)	**−0.051 (−0.0.080, −0.032)**
Outer regional and remote	1103 (1.5)	851 (1.3)	−0.018 (−0.077, 0.040)
**Socio‐economic disadvantage**			
1 – Most disadvantaged	8503 (11.4)	6017 (9.2)	Reference
2	5816 (7.7)	4570 (7.0)	**0.055 (0.023, 0.087)**
3	15 112 (20.2)	12 479(19.2)	**0.081 (0.055, 0.106)**
4	21, 066 (28.1)	18, 512 (28.4)	**0.106 (0.081, 0.131)**
5 – Least disadvantaged	24 447 (32.6)	23 587 (36.2)	**0.154 (0.129, 0.179)**
Mental health diagnoses (% yes)	20 719 (27.7)	19 468 (29.9)	**0.067 (0.052, 0.082)**
**Diagnosis of interest**			
Arthritis/rheumatism (% yes)	11 494 (15.3)	10, 320 (15.8)	−0.002 (−0.019, 0.022)
Back and neck pain (% yes)	17 149 (22.9)	14 721 (22.6)	**−0.031 (−0.047, −0.015)**
Pain related to procedure or injury (% yes)	13, 232 (17.7)	11, 689 (17.9)	−0.001 (−0.017, 0.018)
Migraine and headache (% yes)	8748 (11.7)	8517 (13.1)	**0.057 (0.037‐0.078)**
Other musculoskeletal (% yes)	22, 371 (22.9.)	20 391 (31.3)	0.027 (−0.012, 0.043)
Visceral abdominal or gastrointestinal pain (% yes)	5730 (7.7%)	5281 (8.1)	0.009 (−0.016, 0.034)

*Note*: Bold text indicates significant results. Those in the “not specific” sex categories were coded as missing and excluded from the regression model.

Abbreviations: DVA, Department of Veterans' Affairs.

In addition, when compared to patients without a migraine or headaches diagnosis recorded, the probability of being prescribed codeine increased by 6.0% (95% CI 0.037‐0.078) compared to those that did have a diagnosis recorded. Similarly, those with a mental health diagnosis had 7.0% higher probability of being prescribed codeine (β = 0.067, 95% CI 0.052‐0.082) following rescheduling compared to patient without a mental health diagnosis. Patients with back and neck pain had a 3.1% lower probability of being prescribed codeine after rescheduling (β = −0.031, 95% CI −0.047 to −0.015) compared to patients without this diagnosis.

## 4 DISCUSSION

Rescheduling codeine to prescription‐only did not result in a sustained increase in codeine prescribing nor a change in prescribing of other opioids. These findings are consistent with previous research that reported an 18% reduction in overall opioid dispensing across Australia between 2016‐17 and 2020‐21, and a 30% reduction in the overall volume of opioids dispensed.^34^ Taken together with other studies examining outcomes from codeine rescheduling,^18^, ^35^, ^36^ this suggests that concerns that the scheduling change would lead to an unmanageable burden for the healthcare systems or adverse outcomes for patients^37^ were not realized.

The immediate increase in the monthly rate of codeine prescribing after rescheduling may represent supply for patients who had previously purchased codeine without a prescription. The subsequent reduction in the monthly rate of codeine prescribing without a corresponding increase in prescribing of other opioids suggests codeine prescribing for these patients was not continued in the longer term. These results may be considered in the context of the national education initiatives conducted to promote opioid safety in anticipation of codeine rescheduling. These encompassed various activities, such as workshops focused on codeine rescheduling, online learning modules developed by the Australian and New Zealand College of Anaesthetists to enhance pain management practices, multidisciplinary pain management workshops and webinars organized by Royal Australian College of General Practitioners and allied health support groups.^38^ Additionally, numerous continuing professional development resources were made available through the TGA Codeine Rescheduling Information Hub.^38^ The overall downward trends in opioid use may have also contributed to the trend observed.^34^ Our finding that prescribing of other opioids did not increase aligns with previous research suggesting rescheduling codeine did not increase stronger opioid use.^19^

There were changes in the demographic profile of patients prescribed codeine before and after rescheduling. The small increase in prescribing among females reflects well‐documented gender disparities in opioid utilization.^39^ Australian females may have also had a higher number of pain‐ and non‐pain related GP consultations and therefore greater opportunities to request codeine prescriptions in place of non‐prescription codeine.^40^ These reasons are consistent with Australian females being more likely to have opioids dispensed and in higher quantities than males.^1^, ^41^, ^42^ This highlights the importance of considering sex‐specific issues, such as higher rates of chronic pain among women, when addressing opioid misuse and improving prescription practices.

After the codeine rescheduling, there was a notable shift in prescription for older adults, particularly in the 70‐79 years age group, where the probability of being prescribed codeine increased by 11% compared to individuals aged <30 years. This disproportionally higher rate of prescribing of codeine in older adults as a result of the scheduling change aligns with broader trends where people over 65 years are more likely to be prescribed opioids compared to younger groups,^2^ and also increased in Australian aged care facilities over 2019‐2020.^43^ Given that older people are susceptible to opioid‐related adverse drug events, including nausea, constipation, drowsiness, dizziness, headaches, dry mouth, falls and cognitive impairment, the implications of these prescribing shifts warrant monitoring.^44^

The increased likelihood of codeine prescribing in patients with migraines requires attention. Migraine is one of the top 20 chronic pain conditions treated in general practice.^40^ The high rate of codeine prescription for patients with a migraine diagnosis in primary care seen in this study is consistent with evidence of inappropriate opioid prescribing for migraine in primary care and hospital settings.^45^, ^46^, ^47^, ^48^, ^49^ Australian and international treatment guidelines recommend against using opioids for migraine and instead recommend non‐opioid analgesics and migraine‐specific medications for the treatment of acute and chronic migraine.^50^, ^51^, ^52^, ^53^, ^54^ It is possible that some patients prescribed codeine for migraine after rescheduling were self‐medicating with non‐prescription codeine before rescheduling, hence this increase in prescribed codeine could reflect a continuation rather than inappropriate initiation of codeine. Although we are not able to determine this from our data, there is also the possibility that in some cases, a diagnosis of migraine may actually represent medication overuse headache, which has been associated with chronic codeine use.^55^ Studies have suggested that opioids are prescribed for 38% of patients with migraine in general practice, highlighting that there is a broader issue in improving evidence‐based migraine treatment.^49^

We observed lower likelihood of codeine prescribing for back and neck pain following rescheduling. This may reflect a growing emphasis on non‐opioid management for these conditions, as indicated by recent guidelines and clinical trial results.^56^, ^57^ Despite this positive finding, one in four people prescribed codeine were being treated for back and neck pain, a trend consistent with earlier findings from 2015‐2016 that indicated that 22.1% of patients who received an opioid prescription from their general practitioner did so for treatment of back pain.^6^

## 5 STRENGTHS AND LIMITATIONS

This study's strength lies in the use of a comprehensive general practice dataset spanning 464 general practices and comprising almost 140 000 patients who were prescribed codeine. The 4‐year study period enables a robust examination of codeine prescribing before and after its rescheduling due to the comprehensive capture of both subsidized and non‐subsidized opioid prescriptions, including all low‐dose codeine formulations.

Our study has several limitations to consider. Patient activity was tracked at the practice level in POLAR so patients visiting multiple clinics might not have been identified. However, recent data indicate that more than 80% of Australians see the same GP and a further 15% see a GP in the same practice, indicating that a very small part of the population would see multiple practices, minimizing any likely impact of opioid prescriptions for individuals in our study not being captured.^58^ Moreover, in Victoria, long‐term opioid prescribing is typically managed by a single prescriber under a permit system, which may partially alleviate this issue, although it cannot be entirely eliminated. As with all studies using electronic medical records data, there are known limitations such as incomplete data, the need for thorough cleaning and transformation of data for statistical analysis, and challenges arising from the lack of standardized terminology for diagnoses or clinical findings.^59^ Data quality is reliant on end‐user (ie, clinician) input and, relatedly, documented diagnoses are at the patient level and not linked to individual prescriptions so we are unable to confirm the specific indication for codeine on each occasion. The study was conducted in three PHNs in Victoria and the results may not be generalizable to other parts of Australia. However, rates of opioid prescribing in the three PHNs have been shown to be similar to national rates of opioid prescribing.^34^ Previous research utilizing this data has shown that the demographic characteristics of the patient populations within these practices are consistent with national estimates of comorbidities such as diabetes^60^ and characteristics reported in the national primary care survey data, reducing concerns about generalisability. As use of over‐the‐counter analgesics is not consistently documented in any database, we are not able to examine the transition from over‐the‐counter to prescribed opioids, but the capturing of non‐subsided codeine prescriptions in primary care prescribing data does enable the identification of new patients who were prescribed codeine after the policy change. Codeine rescheduling also coincided with other Victorian initiatives to address opioid misuse, including the introduction of prescription drug monitoring programs, which may in part explain reduced opioid prescribing rates.^27^, ^61^

In conclusion, codeine rescheduling did not result in a sustained increase in codeine prescribing or a change in the prescribing of other opioids. Female sex, older age, migraine diagnosis and comorbid mental health conditions were associated with codeine initiation after the policy change. These findings may inform pain management, where codeine continues to be commonly used in ways that are not consistent with clinical guidelines.

## CONFLICT OF INTEREST STATEMENT

The authors have no conflicts of interest to declare.

## Data Availability

The data that support the findings of this study are available from Outcome Health. Restrictions apply to the availability of these data, which were used under licence for this study.
